# Identification of candidate genes and chemicals associated with osteoarthritis by transcriptome-wide association study and chemical-gene interaction analysis

**DOI:** 10.1186/s13075-023-03164-x

**Published:** 2023-09-25

**Authors:** Lin Mei, Zhiming Zhang, Ruiqi Chen, Zhongyue Liu, Xiaolei Ren, Zhihong Li

**Affiliations:** 1 Department of Orthopedics, The Second Xiangya Hospital, Central South University, Changsha, China; 2 Hunan Key Laboratory of Tumor Models and Individualized Medicine, The Second Xiangya Hospital, Changsha, China

**Keywords:** Osteoarthritis, GWAS, TWAS, CGSEA

## Abstract

**Background:**

Osteoarthritis (OA) is a common degenerative joint disease and causes chronic pain and disability to the elderly. Several risk factors are involved, such as aging, obesity, genetic susceptibility, and environmental factors. We conducted a transcriptome-wide association study (TWAS) and chemical-related gene set enrichment analysis (CGSEA) to investigate the susceptibility genes and environmental factors.

**Methods:**

TWAS analysis was conducted to identify the susceptibility genes by integrating the summary-level genome-wide association study data of knee OA (KOA) and hip OA (HOA) with the precomputed expression weights from the Genotype-Tissue Expression Project (Version 8). The FUSION software was used for both single-tissue and cross-tissue TWAS, which were combined using an aggregate Cauchy association test. The biological function and pathways of the TWAS genes were explored using the Kyoto Encyclopedia of Genes and Genomes (KEGG) and Gene Ontology (GO) databases, and the human cartilage mRNA expression profiles were utilized to validate the TWAS genes. CGSEA analysis was performed to scan the OA-associated chemicals by integrating the TWAS results with the chemical-related gene sets.

**Results:**

There were 44 and 93 unique TWAS genes identified in 7 and 11 chromosomes for KOA and HOA, respectively, fourteen and four of which showed significantly differential expression in the mRNA profiles, such as CRHR1, LTBP1, WWP2, LMX1B, and PTHLH. OA-related pathways were found in the KEGG and GO analysis, such as TGF-beta signaling pathway, MAPK signaling pathway, hyaluronan metabolic process, and chondrocyte differentiation. Forty-five OA-associated chemicals were identified, including quercetin, bisphenol A, and cadmium chloride.

**Conclusions:**

Several candidate OA-associated genes and chemicals were identified through TWAS and CGSEA analysis, which expanded our understanding of the relationship between genes, chemicals, and their impact on OA.

**Supplementary Information:**

The online version contains supplementary material available at 10.1186/s13075-023-03164-x.

## Introduction

Osteoarthritis (OA), a common degenerative joint disease, causes chronic pain and disability in the elderly. According to the data from the Global Burden of Disease project, the age-standardized point prevalence and annual incidence rate of OA were 3754.2 and 181.2 per 100,000 in 2017, with an increase of 9.3% and 8.2% from 1990, respectively [1].

While the pathogenesis of OA is not entirely explained, the risk factors for OA development have been demonstrated in epidemiologic studies, such as age, obesity, ethnicity, family history and genetic factors, and environmental factors [2, 3]. Moreover, genetic factors have been reported to have a significant contribution to knee OA (KOA) and hip OA (HOA), and the heritability has been estimated to be 60% for the HOA and 50.4% for the KOA [4, 5]. Recently, genetic studies have yielded novel insights into the genetic propensity of OA. Several genome-wide association studies (GWAS) have identified more than 100 loci associated with OA [6–10], further elucidating the genetic architecture of OA. However, the specific mechanism between those genetic variants and OA has not been fully investigated. The genetic variants have been demonstrated to have impact on the gene expression to further influence the phenotypes [11, 12], and transcriptome-wide association studies (TWAS) have offered the chance to integrate the summary-level GWAS data with expression quantitative trait loci (eQTL) references to identify the trait-related genes.

Previous studies have identified the OA-associated genes by integrating the TWAS results and mRNA expression profiles for HOA and KOA [13, 14]. However, both studies adopted the skeletal muscle and blood as the eQTL references, which are not the causally relevant tissue of OA. In addition, performing TWAS using the eQTL panels of non-trait-related tissues leads to non-causal hits and dropped out of the real causal ones, and cross-tissue TWAS has been recommended if there is no closely trait-related tissue available [15].

In the present study, we conducted both single-tissue and cross-tissue TWAS using summary-level GWAS data of HOA and KOA to investigate gene-trait associations. Subsequently, biological pathways of the TWAS genes were explored. Finally, chemical-related gene set enrichment analysis (CGSEA) was performed to identify chemicals associated with OA using the Comparative Toxicogenomics Database (CTD).

## Methods

### Summary-level GWAS data

The summary-level GWAS data were downloaded from GeneATLAS (http://geneatlas.roslin.ed.ac.uk/) [16]. In this study, we adopted GWAS summary statistics for HOA (M16 Coxarthrosis, Ncases = 12,868, Ncontrols = 439,396) and KOA (M17 Gonarthrosis, Ncases = 21,918, Ncontrols = 430,346), with a combined sample size of 452,264 individuals of European ancestry from UK Biobank [17]. The summary-level GWAS outcomes were subsequently reformatted into the*.sumstats* format using the *munge_sumstats.py* program from the LD Score regression software package, which was publicly accessible at https://github.com/bulik/ldsc.

### Transcriptome-wide association studies

In this study, we employed the FUSION software (http://gusevlab.org/projects/fusion/) to conduct a summary-based TWAS using LD reference data on built GRCh38 of European populations from the 1000 Genomes project (version 3) downloaded from the Alkes Group website (https://alkesgroup.broadinstitute.org/LDSCORE/GRCh38/) [18]. The eQTL reference panels from the Genotype-Tissue Expression (GTEx) Project (version 8) (https://gtexportal.org/home/datasets) were downloaded from the FUSION website [19].

As there was no causally OA-related tissue reference panel, such as cartilage and chondrocyte, we utilized all GTEx reference panels in the TWAS as recommended [15]. Additionally, to enhance the power of the TWAS, we performed a sparse canonical correlation analysis (sCCA) TWAS with cross-tissue reference panels publicly available on the FUSION website. The single-tissue TWAS and sCCA-TWAS were subsequently merged using an aggregate Cauchy association test (ACAT). The comprehensive analytical methodology of the sCCA-ACAT approach was demonstrated in the previous study (https://github.com/fenghelian/sCCA-ACAT_TWAS) [20].

The TWAS was performed on the autosomal chromosomes with the default settings. A strict Bonferroni-corrected *P*-value (*P*._Bonferroni_) < 0.05 was considered as a threshold for the significant TWAS associations.

### Functional exploration of significant TWAS genes

To investigate the potential biological function of the identified significant TWAS genes, enrichment analysis was conducted using the Kyoto Encyclopedia of Genes and Genomes (KEGG) and Gene Ontology (GO) databases [21, 22]. The KEGG and GO enrichment analysis was performed using the “*clusterProfiler*” R package (R Foundation for Statistical Computing, Vienna, Austria; https://www.R-project.org/).

### Gene expression dataset of cartilage

For KOA, the present study leveraged the gene expression dataset of cartilage obtained from a previous study [23]. This dataset comprised mRNA-sequencing data extracted from knee cartilage tissue of 18 healthy donors and 20 OA patients, characterizing all genes in terms of log2 fold change (FC) and an adjusted *P*-value. To ascertain differentially expressed genes (DEGs), a significance threshold of adjusted *P*-value < 0.05 was applied.

To validate the HOA TWAS genes, a total of 10 pairs of preserved and OA cartilage samples (age ≥ 73) from the Research Arthritis and Articular Cartilage study were included to analyze the gene expression profile of hip joint [24]. We used the GEO2R program in Gene Expression Omnibus database (https://www.ncbi.nlm.nih.gov/geo/) to find out the DEGs, with a significance threshold of *P*-value < 0.05.

### Chemical-gene expression interaction

The CTD (http://ctdbase.org/) is a publicly available online database that provides access to data on chemical-gene interactions, chemical-disease associations, chemical-pathway associations, and chemical-phenotype associations. A flexible tool (CGSEA, https://github.com/ChengSQXJTU/CGSEA) was introduced to screen potential chemicals implicated in complex diseases or traits, and 11,190 chemical-associated gene sets were generated using 1,788,149 annotation terms of chemical-gene pairs acquired from human and mice. The comprehensively analytical methodology was elucidated in the previous study [25].

In the present study, we integrated the TWAS results with the chemical-related gene sets to scan the candidate chemicals associated with OA using the *CGSEA* program with the default settings. The chemicals with both absolute value of normalized enrichment score (|NES|) > 1 and *P*-value < 0.05 were considered the significantly OA-associated chemicals.

## Results

### Transcriptome-wide association studies

**Fig. 1:**
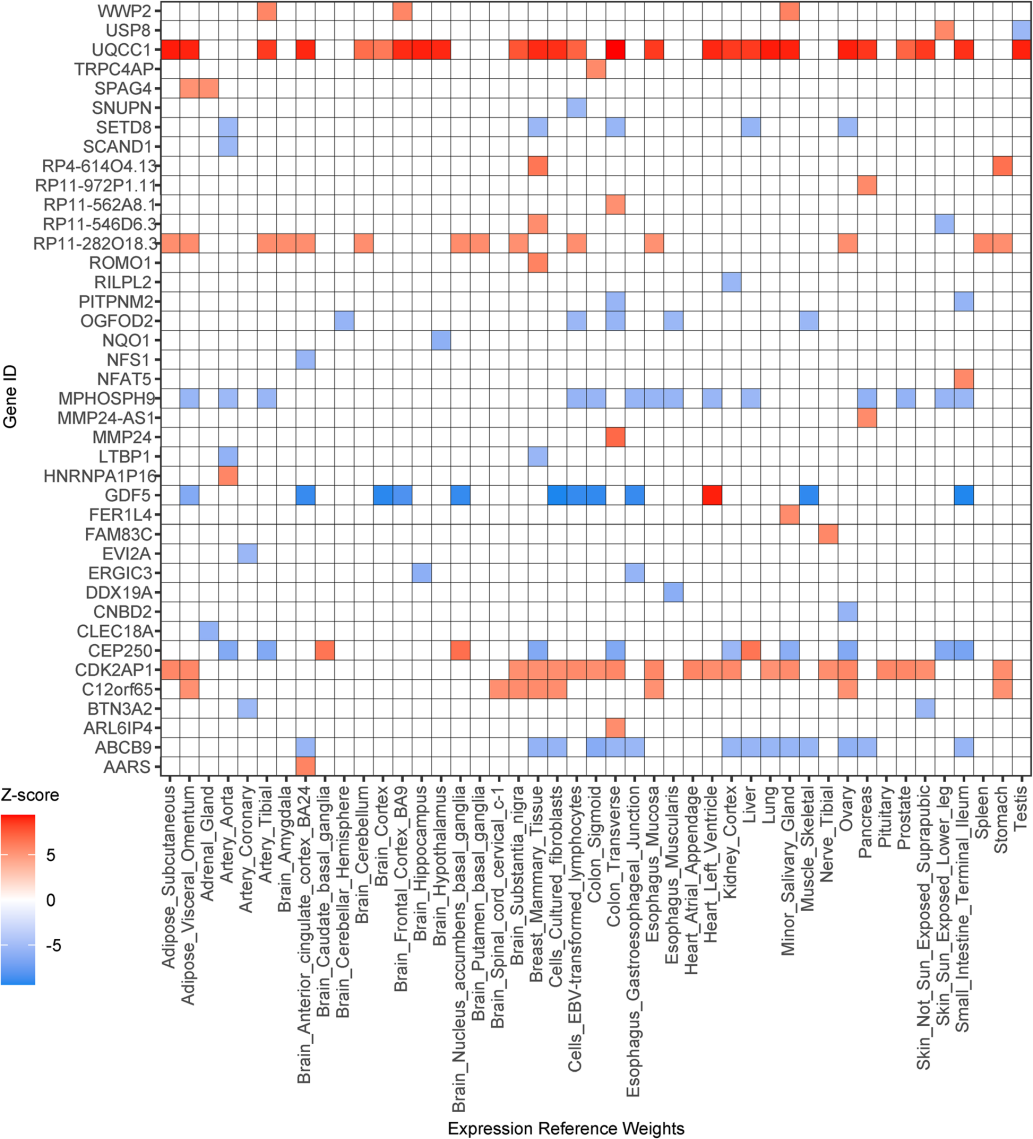
The significant TWAS genes for knee OA with Z-scores across multiple reference panels. White spaces indicated the genes did not pass the significance threshold

**Fig. 2:**
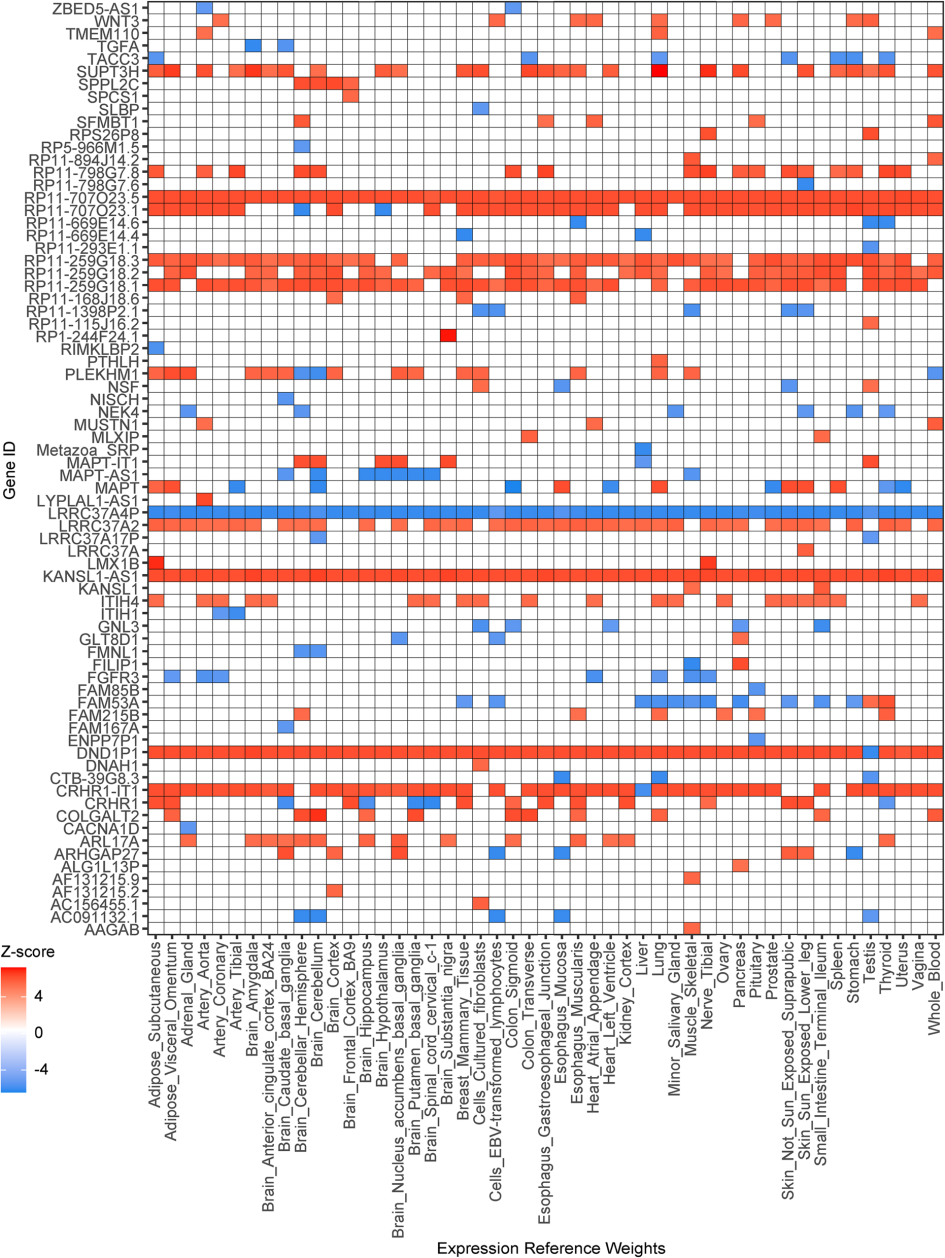
The significant TWAS genes for hip OA with Z-scores across multiple reference panels. White spaces indicated the genes did not pass the significance threshold

**Fig. 3:**
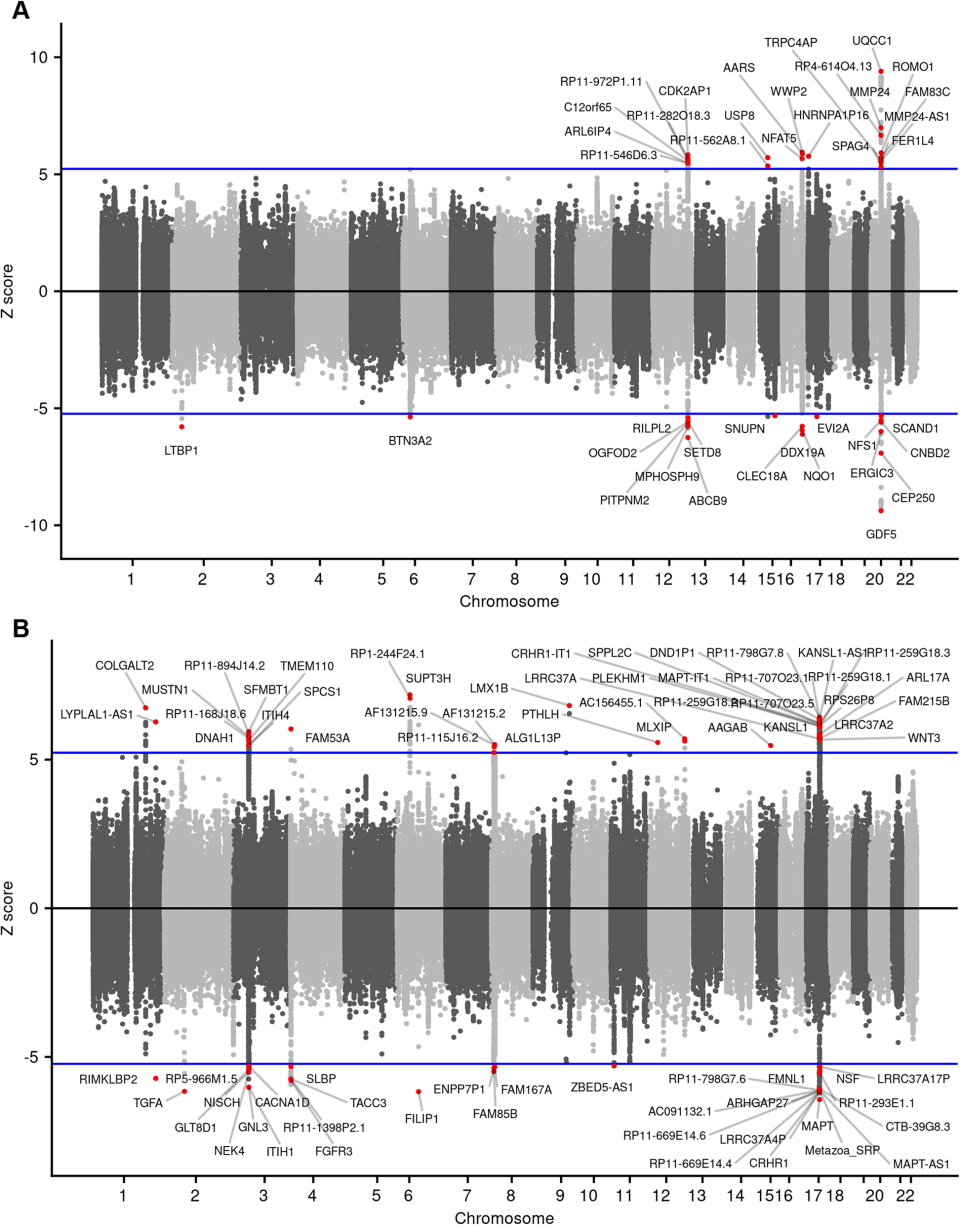
Manhattan plots of the TWAS results. **A** and **B** showed the significant TWAS genes of knee OA and hip OA, respectively, across all autosomes. The blue lines indicated the significance threshold of the TWAS analysis. The labeled feature was the most significant one when there were multiple features with the same ID achieving significance

We identified 170 and 731 significantly associated genes (*P*._Bonferroni_ < 0.05) across multiple eQTL reference panels in the single-tissue TWAS of KOA and HOA, respectively (Figs. 1 and 2, Table S1-2). Amongst those features, 40 and 74 unique genes were identified in 7 and 11 chromosomes for KOA and HOA, respectively (Fig. 3). While most features showed the consistent expression patterns across expression panels, there were 4 and 10 genes with inconsistent direction of effect (Figs. 1 and 2). Additionally, we conducted cross-tissue TWAS through the sCCA + ACAT approach to improve the power of single-tissue TWAS and found another 9 and 19 significant features (*P*._Bonferroni_ < 0.05) for KOA and HOA, respectively (Table 1, Table S3-4).

**Table 1:** The additional significantly associated genes identified through the ACAT approach

OA	Symbol	*P*.ACAT	*P*.Bonferroni
Knee	AC005152.3	1.71E − 06	4.90E − 02
	AL450226.2	1.76E − 07	5.04E − 03
	COG8	1.30E − 07	3.74E − 03
	CYB5B	3.14E − 07	8.98E − 03
	HIC1	1.11E − 07	3.18E − 03
	RP1-221C16.8	9.64E − 07	2.76E − 02
	RP11-234K24.3	5.32E − 07	1.52E − 02
	RP11-419C5.2	1.73E − 06	4.94E − 02
	RP11-667K14.3	3.33E − 08	9.53E − 04
Hip	ALAS1	2.49E − 07	7.13E − 03
	ALG1L11P	1.17E − 06	3.36E − 02
	CLIP1	7.76E − 08	2.22E − 03
	DIABLO	3.26E − 07	9.33E − 03
	FAM86B3P	6.68E − 07	1.91E − 02
	FAM90A10P	4.67E − 07	1.34E − 02
	GLYCTK-AS1	1.01E − 06	2.89E − 02
	ITIH3	1.18E − 08	3.39E − 04
	MALAT1	8.25E − 07	2.36E − 02
	NT5DC2	1.61E − 06	4.61E − 02
	PBRM1	3.06E − 07	8.76E − 03
	RP11-481A20.4	1.36E − 06	3.90E − 02
	RP11-67K19.3	2.18E − 07	6.24E − 03
	RP5-966M1.7	7.71E − 07	2.21E − 02
	RPS7P11	5.52E − 07	1.58E − 02
	SETD1B	8.67E − 08	2.48E − 03
	SSSCA1-AS1	2.20E − 11	6.30E − 07
	TNC	3.00E − 08	8.58E − 04
	TNNC1	1.17E − 07	3.35E − 03

*OA* Osteoarthritis, *ACAT* Aggregate Cauchy association test, *P.ACAT P*-value from ACAT analysis, *P.Bonferroni* Bonferroni-corrected *P*-value from ACAT analysis

### Functional annotation of TWAS genes

**Fig. 4:**
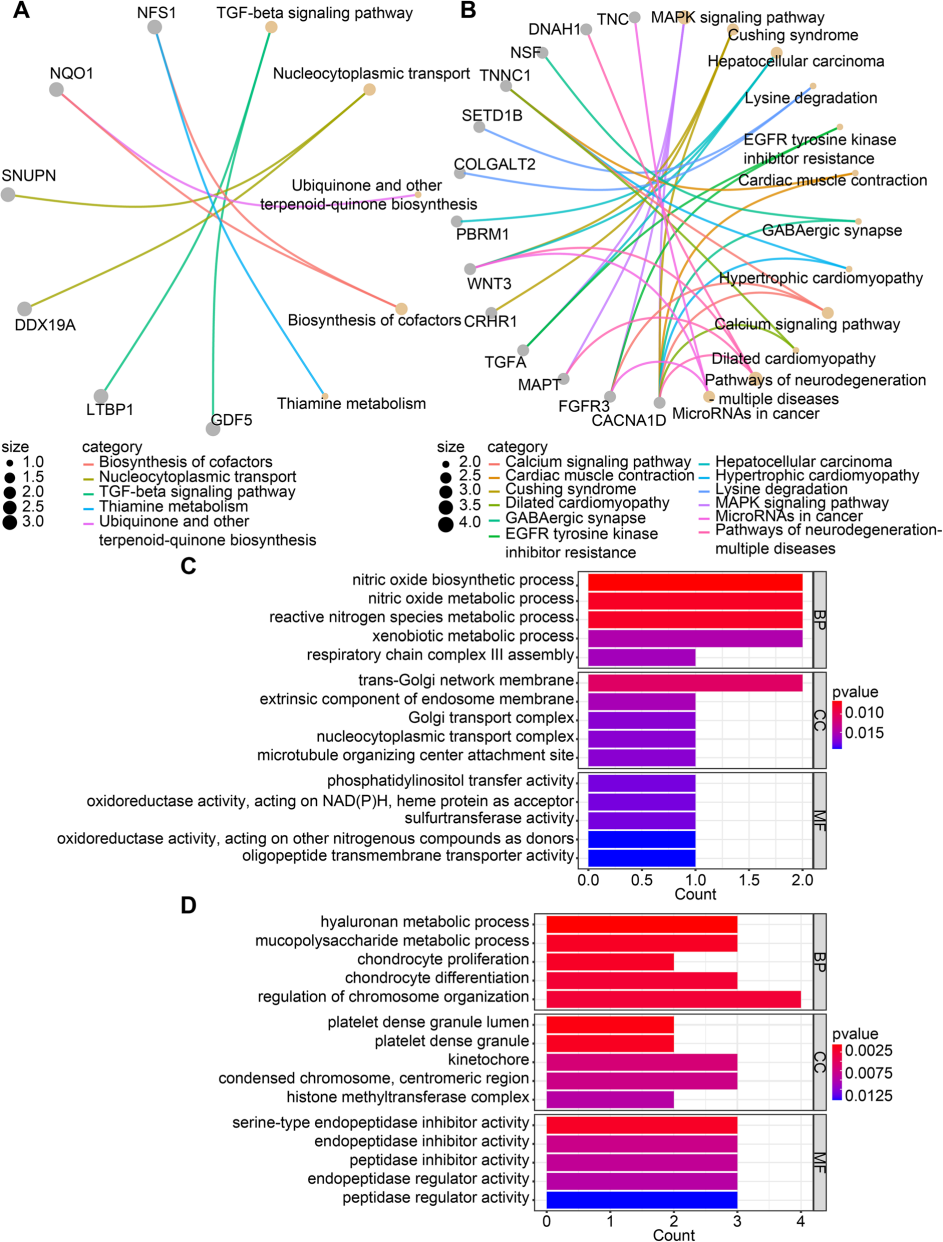
The significant KEGG categories and top five GO terms of TWAS genes. **A** and **B** showed the significant KEGG categories for knee OA and hip OA, respectively. **C** and **D** showed the top five GO terms for knee OA and hip OA, respectively

KEGG and GO analyses were applied to explore the potential biological function of the TWAS genes, which were identified by either single-tissue or cross-tissue TWAS analysis. There were 4 and 12 KEGG categories with *P*-value < 0.05 for KOA and HOA, respectively (Fig. 4 A, B), including TGF-beta signaling pathway and MAPK signaling pathway, which played an important role in OA [26]. The TWAS genes were subjected to GO analysis as well (Table S5-6), and Fig. 4 C, D shows the top five GO terms in the biological process, cellular component, and molecular function category. Particularly, we found some OA-related biological processes in the biological process category, such as hyaluronan metabolic process, chondrocyte differentiation, chondrocyte proliferation, and mucopolysaccharide metabolic process.

### Shared genes in mRNA expression profiling

**Fig. 5:**
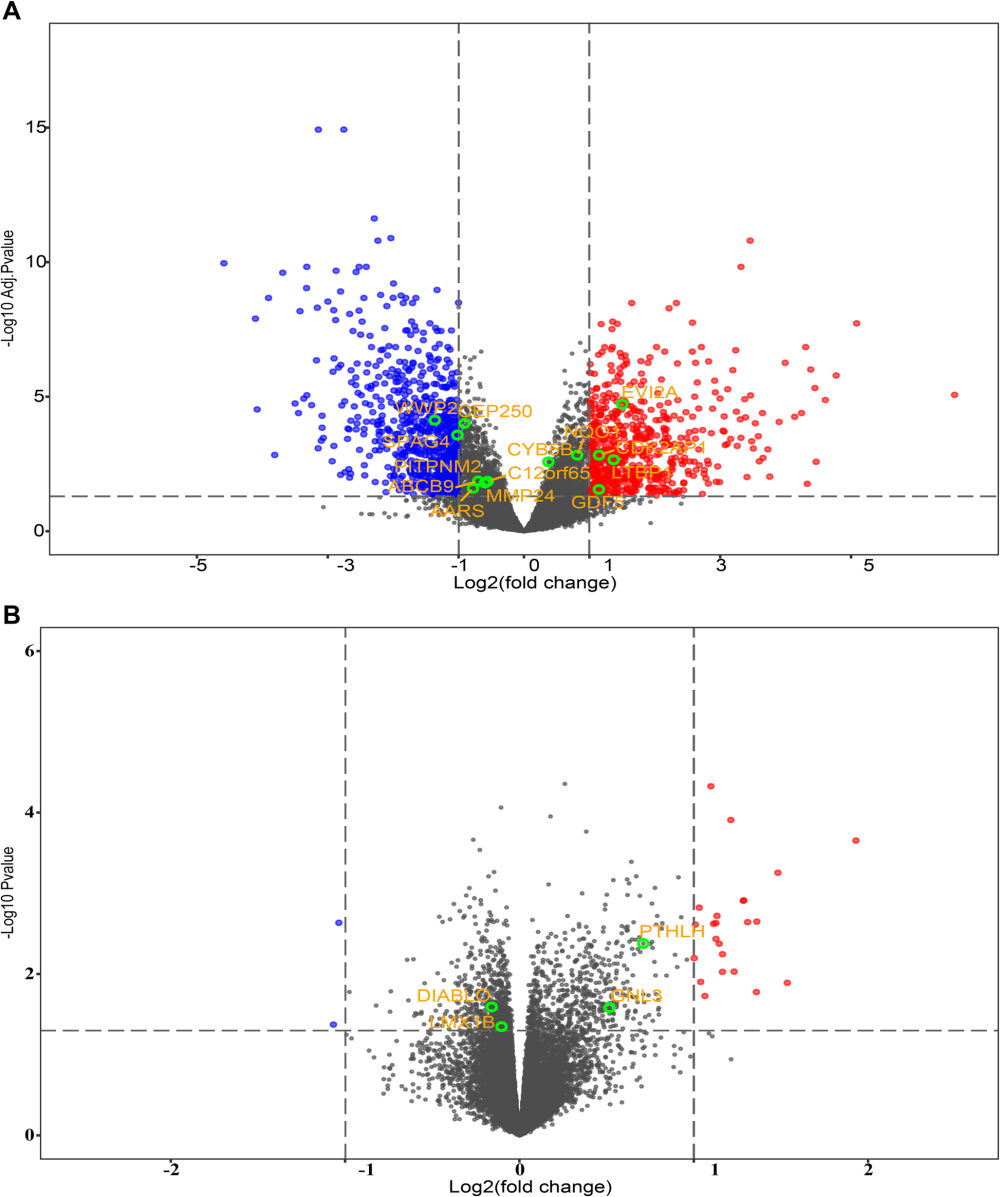
The shared significant genes identified in both TWAS analysis and cartilage mRNA expression profiles. **A** for knee OA and **B** for hip OA

To validate the significant TWAS features, we utilized the cartilage mRNA expression profiling from previous studies [23, 24]. In the case of KOA and HOA, a total of 14 and 4 TWAS genes, respectively, were observed among the DEGs in the mRNA expression analysis (adjusted *P*-value < 0.05) (Fig. 5). Notably, among these shared genes, six genes for KOA exhibited a |log2FC|> 1, as shown in Table 2.

**Table 2:** The shared genes between significant TWAS genes and DEGs with |log2FC|> 1

OA	Symbol	log2_FC	Adjusted_*P*-value
Knee	CDK2AP1	1.15	1.49E − 03
	EVI2A	1.50	1.93E − 05
	GDF5	1.14	2.81E − 02
	LTBP1	1.37	2.21E − 03
	SPAG4	− 1.02	2.68E − 04
	WWP2	− 1.36	7.46E − 05

*TWAS* Transcriptome-wide association study, *DEGs* Differentially expressed genes, *OA* Osteoarthritis, *FC* Fold change

### CGSEA of the TWAS genes

We conducted CGSEA analysis to identify the OA-associated environmental factors using the significant TWAS genes with the *Z*-scores in the single-tissue TWAS (Table 3). For KOA, we identified the 5 enriched chemicals. Meanwhile, there were 45 significantly enriched chemicals for HOA (Table S7), such as quercetin, bisphenol A, cadmium chloride, and mercuric chloride.

**Table 3:** The top five significantly OA-associated chemicals identified through CGSEA analysis

OA	Chemical Name	MeSH® ID	NES	*P*-value
Knee	Acetaminophen	D000082	15.90	9.99E − 04
	Tetrachlorodibenzodioxin	D013749	37.22	9.99E − 04
	Aflatoxin B1	D016604	65.49	9.99E − 04
	Bisphenol A	C006780	6.69	2.00E − 03
	Cyclosporine	D016572	13.42	3.00E − 03
Hip	PCB 180	C410127	118.04	9.99E − 04
	Mercuric Chloride	D008627	76.38	9.99E − 04
	Atrazine	D001280	64.67	9.99E − 04
	Quercetin	D011794	61.42	9.99E − 04
	Genistein	D019833	56.48	9.99E − 04

*OA* Osteoarthritis, *CGSEA* Chemical-related gene set enrichment analysis, *NES* Normalized enrichment score

## Discussion

The present study identified 49 and 93 significantly associated genes of KOA and HOA, respectively, through single-tissue and cross-tissue TWAS, and the biological function and pathways of those signals were further investigated using KEGG and GO databases. We used the gene expression profiling of cartilage to validate the TWAS results.

More than 100 independent risk variants of OA have been reported in previous GWAS studies. While several the significant TWAS genes overlapped with the effector genes or reasonable candidate genes in the GWAS analysis, such as GDF5, USP8, TNC, FGFR3, LTBP1, and UQCC1, we unraveled several novel potential risk genes by TWAS analysis, which also showed significantly differential expression in the cartilage mRNA expression profiling, such as EVI2A, FMNL1, and AARS. Actually, studies demonstrated that some of the TWAS genes were involved in the OA-related biological function, such as GDF5 [27–29], WWP2 [30, 31], and MALAT1 [32–34], while several TWAS genes have not been fully investigated in OA. For example, the MLXIP gene, also known as MAX-interacting protein 1 or MIP1, encodes a protein that interacts with the MAX transcription factor, which plays a critical role in the regulation of gene expression. Studies have shown that the MLXIP gene is involved in various biological processes, including glucose metabolism [35, 36], lipid metabolism [37–39], and cellular senescence [40], which have also been implicated in the pathogenesis of osteoarthritis.

Additionally, by integrating the TWAS results with the chemical-related gene sets, we found a total of 45 unique OA-associated chemical substances. Among the identified chemicals, quercetin was extensively studied in the field of OA and alleviated OA through its multiple biological functions, including suppression of the inflammation and cartilage degeneration, pain relief, and attenuation of oxidative stress, ER stress, and associated apoptosis [41–45]. Clinical studies also support the protective effect of quercetin supplement, which could significantly improve the joint function and collagen II synthesis/degradation balance [46]. There were some hazardous chemicals among the identified OA-associated ones. For instance, bisphenol A was detected not only in the serum of the OA patients, but also in the synovial fluid of knee replacement patients, and exhibited a concentration-dependent antagonistic effect on the protective actions of E2 on chondrocyte, which decreased the NF-kappaB activation and MMP1 expression [47]. In addition, the exposure of cadmium chloride could reduce the chondrocyte cell viability, increase the expression of the catabolic markers (MMP13, MMP9, MMP3, MMP1) and inflammatory markers (IL-1β and IL-6), and activate the expression of the cartilage extracellular matrix genes (aggrecan and collagen II), and the cadmium contributed the cartilage loss by activating the interleukins through the reactive oxygen species [48]. Clinical findings supported the harmful effect of cadmium, and cadmium exposure through smoking was positively correlated with the severity of OA [49].

There were several limitations of the present study. First, the lack of causally OA-related tissues could reduce the power of the TWAS analysis, while we utilized both single-tissue and cross-tissue TWAS approaches to address this issue. Second, we employed the bioinformatic analysis to explore the OA-related candidate genes and chemicals, and the biological function of TWAS genes and related chemicals needed to be further investigated through biological experiments and clinical observation. Third, the summary-level GWAS data were exclusively derived from the European population, and caution should be exercised when extrapolating the results to other populations.

## Conclusions

In summary, we identified multiple OA-associated genes and chemicals by performing the TWAS and CGSEA analysis and yielded novel insights into the relationship between genes, chemicals, and their impact on OA.

### Supplementary Information

**Additional file 1.**

## Data Availability

The software and datasets used and/or analyzed during the current study are available from FUSION (http://gusevlab.org/projects/fusion/); the Comparative Toxicogenomics Database (http://ctdbase.org/); CGSEA (https://github.com/ChengSQXJTU/CGSEA); Gene ATLAS (http://geneatlas.roslin.ed.ac.uk/), key: clinical_c_M17 and clinical_c_M16; the Alkes Group (https://alkesgroup.broadinstitute.org/LDSCORE/GRCh38/); LD Score regression software packages (https://github.com/bulik/ldsc); Gene Expression Omnibus database (https://www.ncbi.nlm.nih.gov/geo/).

## Abbreviations

OA: Osteoarthritis
eQTL: Expression quantitative trait loci
GWAS: Genome-wide association study
TWAS: Transcriptome-wide association studies
sCCA: Sparse canonical correlation analysis
ACAT: Aggregate Cauchy association test
GTEx: Genotype-Tissue Expression
GO: Gene Ontology
KEGG: Kyoto Encyclopedia of Genes and Genomes
CGSEA: Chemical-related gene set enrichment analysis
CTD: Comparative Toxicogenomics Database
DEGs: Differentially expressed genes
